# Correlations between fMRI activation and individual psychotic symptoms in un-medicated subjects at high genetic risk of schizophrenia

**DOI:** 10.1186/1471-244X-7-61

**Published:** 2007-10-29

**Authors:** Heather C Whalley, Viktoria-Eleni Gountouna, Jeremy Hall, Andrew McIntosh, Marie-Claire Whyte, Enrico Simonotto, Dominic E Job, David GC Owens, Eve C Johnstone, Stephen M Lawrie

**Affiliations:** 1 Division of Psychiatry, School of Molecular and Clinical Medicine, University of Edinburgh, Edinburgh, Scotland, UK.

## Abstract

**Background:**

It has been proposed that different types of psychopathology in schizophrenia may reflect distinguishable pathological processes. In the current study we aimed to address such associations in the absence of confounders such as medication and disease chronicity by examining specific relationships between fMRI activation and individual symptom severity scores in un-medicated subjects at high genetic risk of schizophrenia.

**Methods:**

Associations were examined across two functional imaging paradigms: the Hayling sentence completion task, and an encoding/retrieval task, comprising encoding (at word classification) and retrieval (old word/new word judgement). Symptom severity was assessed using the positive and negative syndrome scale (PANSS). Items examined were hallucinations, delusions, and suspiciousness/persecution.

**Results:**

Associations were seen in the anterior middle temporal gyrus in relation to hallucination scores during the sentence completion task, and in the medial temporal lobe in association with suspiciousness/persecution scores in the encoding/retrieval task. Cerebellar activation was associated with delusions and suspiciousness/persecution scores across both tasks with differing patterns of laterality.

**Conclusion:**

These results support a role for the lateral temporal cortex in hallucinations and medial temporal lobe in positive psychotic symptoms. They also highlight the potential role of the cerebellum in the formation of delusions. That the current results are seen in un-medicated high risk subjects indicates these associations are not specific to the established illness and are not related to medication effects.

## Background

Schizophrenia is characterised by the presence of positive psychotic symptoms, particularly hallucinations and delusions. The form and content of these symptoms can vary considerably between patients and it has been suggested that different types of psychopathology may reflect distinguishable pathological processes [1]. A number of approaches have been taken to examine such associations [2], including scanning patients with specific symptoms against a suitable control group, or examining patients during the active experience of individual symptoms. Neural substrates implicated in positive psychotic symptoms have included medial temporal lobe (MTL) structures [3-5], lateral temporal cortex [6], and inferior frontal cortex [6-8].

Those studies that have further split positive psychotic symptoms have implicated the lateral temporal cortex in auditory hallucinations, primarily auditory and language related areas in the superior temporal gyrus [9-15]. However, other regions have also been reported to be associated with auditory hallucinations including medial and anterior temporal lobe [10,13,14,16], medial and lateral prefrontal regions [10-12,14,16,17], and cerebellum [12,13]. Far fewer studies have examined the neural correlates of delusions and results are at present inconclusive [18-20].

Such studies are however often small and confounded by antipsychotic medication effects. An alternative approach is to examine a larger group of subjects who are perhaps less severely affected, better able to co-operate with testing, and less likely to be prescribed antipsychotic medication. Individual psychotic symptoms have been noted in the general population (~10%) [21], and have been shown to be more prevalent in high risk samples (~40%) [22]. In the Edinburgh High Risk Study psychotic symptoms were associated with, although not necessarily followed by, subsequent conversion to schizophrenia (as only about 25% converted). Examining relationships between brain activation and the severity of sub-diagnostic psychotic symptoms in high risk subjects in the absence of anti-psychotic medication effects may improve our understanding of the biological basis of the early stages of schizophrenia.

In the current study our aim was to map the neural underpinnings of hallucinations and delusions. Current neuropsychological models of these symptoms stress the importance of disruptions in language processing and mnemonic functions [1,23]. The tasks used in the current study (a verbal fluency task and a verbal encoding/retrieval task) were chosen on the basis of their involvement in these networks and cognitive processes. Based on the schizophrenia literature we hypothesised significant correlations between the severity of hallucinations and delusions and activation in MTL structures, and between hallucination scores and activation in lateral temporal regions.

## Methods

### Subject details

The Edinburgh High Risk Study (EHRS) examined young adults aged 16–24 years at ascertainment (1995–1999) at enhanced genetic risk of schizophrenia (with two or more affected first or second degree relatives) over the period at which they are at greatest risk of becoming ill, in comparison with matched healthy controls with no family history of psychotic illness [22,24,25]. All subjects were antipsychotic naïve throughout the study. This report presents results obtained during the second phase of the study (1999–2004) when fMRI was introduced into the protocol. All subjects were supplied with detailed written information regarding the study and provided written informed consent. The study was approved by the Psychiatry and Clinical Psychology subcommittee of the Lothian research ethics committee. Since this was a within group correlation study the healthy control data were not included, further details are available in earlier publications [26,27].

A total of 96 high risk subjects underwent an fMRI scan which included two functional imaging paradigms; the Hayling sentence completion test (experiment 1) and the encoding/retrieval task (experiment 2). Three subjects were excluded from further analysis due to minor vascular malformations and a temporal lobe cyst. For experiment 1 a further four subjects were excluded due to movement artefact leaving 89 fMRI scans. For experiment 2, seven scans were excluded (five due to excessive movement and two due to loss of behavioural data) leaving 86 fMRI scans. These samples included 69 high risk subjects previously reported [26,27], with an additional 20 subjects representing the completed cohort. There was an overlap of 83 subjects who provided usable scans for both paradigms. For the sentence completion task the mean age was 26.2 years (s.d. 3.3), comprising 42 males and 47 females. For the encoding/retrieval task the mean age was 26.1 years (s.d. 3.3), comprising 41 males and 45 females. The majority of subjects were working or in full time higher education [22]. None of the subjects were on anti-psychotic medication, seeking treatment or saw themselves as unwell, nor did they fulfil diagnostic criteria for any psychiatric disorder.

The severity of positive psychotic symptoms was determined using the positive and negative syndrome scale (PANSS) [28] conducted by two experienced clinicians who had previously undergone the PANSS training programme and had reached the set standard of reliability [29]. Since the primary interest of the current report were activations associated with delusions and hallucinations, the PANSS scores for these measures (first, third, and sixth items on the positive scale representing delusions, hallucinations and suspiciousness/persecution) were entered into further analysis. The score for suspiciousness/persecution was selected along with the score for delusions as it is considered to reflect a paranoid element.

### Scanning procedure

Imaging was carried out at the Brain Imaging Research Centre (BIRC) for Scotland on a GE 1.5 T Signa scanner (GE Medical, Milwaukee, USA). The imaging protocol consisted of a localiser scan, followed by a T2-weighted fast spin-echo sequence, and a structural T1 weighted sequence followed by the functional imaging paradigms. For experiment 1 axial gradient-echo planar images (EPI) (TR/TE = 4000/40 ms; matrix = 64 × 128; field of view (fov) 220 × 440 mm) were acquired continually. Thirty eight contiguous 5-mm slices aligned to the anterior and posterior commissure were acquired within each TR period. Each acquisition was run for 204 volumes, of which the first four volumes were discarded. For experiment 2 functional images were again collected using an EPI sequence (TR/TE = 2000/40 ms; matrix = 64 × 64; field of view (fov) 220 × 220 mm). Twenty-four contiguous 5 mm axial (horizontal) slices were collected at an oblique angle aligned with the anterior and posterior commissure. Data were acquired during 2 sessions, consisting of 104 volumes for the first (word classification) and 204 volumes for the second session (word retrieval), for each session the first four volumes were discarded.

### Experiment 1: Hayling sentence completion task (blocked design)

Participants performed the verbal initiation section of the Hayling sentence completion test in the scanner as described previously [26]. Subjects were shown sentences with the last word missing and were asked to silently think of an appropriate word to complete the sentence and press a button when they had done so. Sentences were presented in blocks of fixed difficulty, determined by the range of suitable words suggested by the sentence context, of which there were four levels. Each block lasted 40 seconds and included 8 sentences. Sentences were presented for a period of 3 seconds followed by a fixation cross for 2 seconds and subjects could respond at any time until the next sentence appeared. The rest condition consisted of viewing a screen of white circles on a black background for 40 seconds. The order of the blocks was pseudo-random, and each block was repeated four times. Immediately after scanning, subjects were given the same sequence of sentences on paper and requested to complete each sentence with the word they first thought of in the scanner. 'Word appropriateness' scores were determined from the word frequency list of sentence completion norms [30].

### Experiment 2: verbal encoding and retrieval (event-related)

During the word classification, or 'encoding', part of the task single words were presented in the scanner and subjects were asked to classify them as 'living' or 'non-living' by pressing a button. A total of 36 words were presented, 18 referring to living things and 18 to non-living things. During the 'retrieval' part of the task single words were presented randomly selected from those shown during the word classification condition along with matched similar new words and subjects were requested to classify them as either 'new' or 'old' words and signify their response by pressing a button. In total 72 words were presented, 36 words which were presented previously (old) intermixed with another 36 matched new words. For more detail see previous study [27]. Stimuli were presented for 2 seconds, followed by a variable fixation period of 2–10 seconds. Responses could be made at any time during presentation and subsequent fixation period by pressing a button. Both parts of the task were preceded by a practice session with feedback.

### Image processing and analysis

Scan analysis was performed using the standard SPM approach in SPM2 [31] running in Matlab (The MathWorks, Natick, MA, USA). Briefly, EPI volumes were realigned to the mean volume in the series, normalised to a study specific EPI template (comprising 121 participants of the EHRS) and spatially smoothed with a 6 × 6 × 6 mm^3 ^FWHM Gaussian filter.

#### First level analysis

Statistical analysis was performed using the general linear model approach as implemented in SPM. Estimates of head movement from the realignment stage of pre-processing were included as additional regressors in the first level analysis. For experiment 1 contrasts were constructed to examine all four sentence completion conditions versus rest, and a parametric contrast which examines increasing activation with increasing difficulty. For the word classification condition of experiment 2 all items were treated as equal and entered into a single regressor model. One contrast of interest was generated examining word classification versus baseline experimental activation. For the retrieval condition three regressors were modelled, correct old words (correct recognition), correct new words (correct rejection) and incorrect words (further splitting of incorrect old and incorrect new was not considered appropriate due to insufficient events in each category). For the purposes of this study contrast images were constructed to examine correct recognition versus baseline experimental activation and correct rejection versus baseline experimental activation.

#### Second level analysis

First level contrast images for each subject were then entered into a second level multiple regression analyses. For each of the five contrasts of interest a single model was used to examine correlations with the selected scores from the PANSS. The three symptom dimensions were modelled simultaneously within one design matrix hence variability common to two or more symptom dimensions is not reflected in the correlation values. In other words, the analysis performed represents, for example, the relationship between delusion severity and fMRI activation that is not accounted for by either suspiciousness or hallucination severity.

Statistical maps were thresholded at a level of p = 0.005 uncorrected, and regions were considered significant at p < 0.05 cluster level corrected for multiple comparisons as performed in SPM. Where significant relationships were found, data were extracted for each cluster (at the maximum voxel) and entered into SPSS (version 12) where a linear regression analysis was performed to check that the standardised residuals were normally distributed and to determine correlation coefficients between the individual symptom severity score and the degree of activation. All p values quoted in the text are at the corrected cluster level as performed in SPM. We would like to state however that positive and negative correlations were examined for each of the five contrasts separately (hence uncorrected voxel-wise p values are one-tailed) and correction for the number of these comparison was not performed. Since we had prior hypotheses regarding temporal lobe involvement in hallucinations we imposed an anatomically defined bilateral temporal lobe small volume correction (SVC) for the examination of correlations with the hallucinations measure [32]. Co-ordinates were converted from MNI (Montreal Neurological Institute) to Talairach co-ordinates using a non-linear transformation [33].

## Results

### Symptom scores and behavioural measures

**Figure 1:**
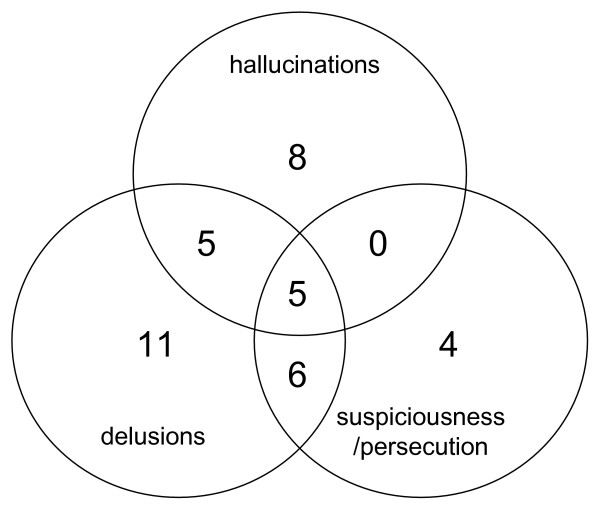
**Distribution of symptom dimensions in high risk individuals**. Values represent the number of subjects with PANSS scores >2 for each symptom type examined. Pair-wise correlation values between the symptom dimensions were as follows: Delusions × hallucinations r = 0.34 (p < 0.01); delusions × suspiciousness/persecution r = 0.48 (p < 0.01); hallucinations × suspiciousness/persecution r = 0.32 (p < 0.01).

The scores for all positive psychotic symptoms as measured by the PANSS are described in Table 1. The distribution of the three symptom dimensions examined in the current study are presented in Figure 1. Pair-wise correlations between the symptom scores are detailed in the figure legend. Behavioural measures (Table 2) indicated subjects were performing both tasks appropriately in the scanner and there were no significant correlations between these measures and any of three symptom severity scores. Previous analysis comparing high risk subjects to normal controls indicated no significant impairment in performance for either of the two tasks [26,27].

**Table 1:** PANSS measures for high risk subjects

	Sentence completion (n = 89)		Encoding/retrieval (n = 86)	
	Mean (SD)	Number of subjects with score > 2	Mean (SD)	Number of subjects with score > 2

P1 delusions	1.51 (0.85)	27	1.52 (0.86)	27
P2 conceptual disorganisation	1.10 (0.40)	6	1.10 (0.41)	6
P3 hallucinations	1.31 (0.67)	18	1.31 (0.67)	17
P4 excitement	1.07 (0.30)	5	1.07 (0.30)	5
P5 grandiosity	1.07 (0.25)	6	1.07 (0.26)	6
P6 suspiciousness/persecution	1.28 (0.71)	15	1.29 (0.72)	15
P7 hostility	1.08 (0.35)	5	1.08 (0.35)	5

PANSS positive total	8.39 (2.4)	-	8.43 (2.4)	-

**Table 2:** Behavioural measures

Hayling sentence completion task		
	Word appropriateness Mean (s.d.)	Reaction time (ms) Mean (s.d.)

Low constraint	6.4 (0.97)	2526 (597)
Medium low constraint	3.3 (0.58)	2427 (621)
Medium high constraint	2.0 (0.37)	2300 (635)
High constraint	1.1 (0.09)	2309 (634)

Encoding/retrieval task		

	Word classification Mean (s.d.)	Word retrieval Mean (s.d.)
Total number correct (out of 36 and 72 stimuli respectively)	35 (1.3)	53* (8.2)
Total number incorrect	0.3 (0.6)	14.2 (5.1)
Reaction time	1037 (440)	1308 (388)

* for correct recognition, total correct = 25 (5.0), for correct rejection, total correct = 28 (5.5)

### Group activation maps

**Figure 2:**
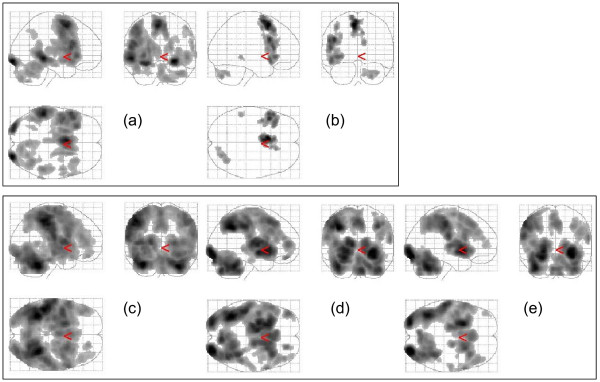
**Within group activation maps for sentence completion task (a, b) and verbal encoding/retrieval task (c-e)**. For the Hayling sentence completion test, contrasts shown are (a) sentence completion versus rest and (b) the parametric contrast representing increasing activation with increasing difficulty. For the encoding/retrieval task, contrasts shown are (c) word classification versus experimental baseline, (d) correct recognition versus experimental baseline, and (e) correct rejection versus experimental baseline. For illustration purposes maps are thresholded at T = 4.5, 4, 6, 5, 5 respectively, extent threshold = 50 voxels. Left hemisphere shown on left of image.

For both experiments the within group maps for all contrasts of interest are shown in a Figure 2. These maps indicated regions commonly activated in these types of tasks and are consistent with previous results [26,27]. For sentence completion versus rest the main areas of activation were the left precentral gyrus, inferior frontal gyrus, medial/superior frontal gyrus, middle/superior temporal gyrus, cerebellum and occipital lobes bilaterally. The parametric contrast indicated increasing activation with increasing difficulty in left superior/medial frontal gyrus, inferior frontal gyrus and cerebellum.

For the encoding/retrieval task, word classification versus baseline experimental activation revealed large areas of activation encompassing bilateral inferior frontal gyrus, superior/medial frontal gyrus, insula, parietal lobe and cerebellum, along with basal ganglia, thalamus and hippocampus. Correct recognition and correct rejection versus experimental baseline revealed similar areas of activation including lateral and superior/medial frontal gyri, parietal lobe, insula, basal ganglia, thalamus and cerebellum. Activation was also observed in the hippocampus at its superior border although this formed part of a larger cluster encompassing basal ganglia and thalamus.

### Correlation with symptom severity

#### Experiment 1: Hayling sentence completion task

**Figure 3:**
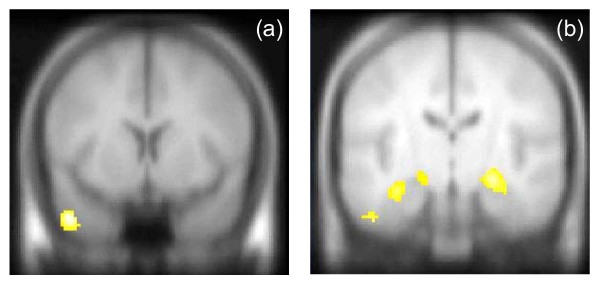
**Correlations with symptom scores in hypothesised regions**. (a) significant positive correlation between severity of hallucinations and activation in left middle temporal gyrus for sentence completion task, (b) significant negative correlation between severity of suspiciousness/persecution score and activation in bilateral MTL for the retrieval task. Maps thresholded at p = 0.005, K_E _= 300 voxels. Left hemisphere shown on left of image

For sentence completion versus rest there was a positive correlation between activation in the left anterior middle temporal gyrus and the hallucinations score (p = 0.01, small volume correction for temporal lobes), see Figure 3a, Table 3.

**Table 3:** Correlation between fmri activation and PANSS scores

Sentence completion task: Sentence completion versus rest								
	+ve correlation				-ve correlation			

	P value (Z score;K_E_)	Correlation*	Co-ordinates	Region	P value (Z score;K_E_)	Correlation*	Co-ordinates	Region

Delusions	n/s	-	-	-	n/s	-	-	-
Hallucinations	0.01^# ^(4.01; 371)	0.41	-48 6 -29	L middle temporal gyrus (BA 21)	n/s	-	-	-
Suspiciousness/persecution	n/s		-	-	0.002 (4.45; 702)	-0.46	0 -74 -35	L posterior lobe of cerebellum

Sentence completion task: Parametric contrast								

Delusions	0.04 (3.83; 337)	0.40	6 -48 -35	R posterior lobe of cerebellum	n/s	-	-	-
Hallucinations	n/s	-	-	-	n/s	-	-	-
Suspiciousness/persecution	n/s	-	-	-	n/s	-	-	-

Verbal encoding: Word classification versus experimental baseline								

	+ve correlation				-ve correlation			

	P value (Z score;K_E_)	Correlation*	Co-ordinatess	Region	P value (Z score;K_E_)	Correlation*	Co-ordinates	Region
Delusions	0.001 (4.28; 1097)	0.45	22 -62 -35	R posterior lobe of cerebellum	n/s	-	-	-
	0.023 (3.32; 664)	0.35	-26 -89 -22	L posterior lobe of cerebellum				
Hallucinations	n/s	-	-	-	0.041 (3.51; 582)	-0.38	44 -70 27	R angular gyrus (BA39)
Suspiciousness/persecution	n/s	-	-	-	n/s	-	-	-

Verbal retrieval: Correct recognition versus experimental baseline								

Delusions	n/s	-	-	-	n/s	-	-	-
Hallucinations	n/s	-	-	-	n/s	-	-	-
Suspiciousness/persecution	n/s	-	-	-	n/s	-	-	-

Verbal retrieval: Correct rejection versus experimental baseline								

Delusions	0.036 (3.68; 856)	0.39	22 -12 -6	R lentiform nucleus	n/s	-	-	-
Hallucinations	n/s	-	-	-	n/s	-	-	-
Suspiciousness/persecution	n/s	-	-	-	0.013 (3.98; 1083)	-0.42	22 -12 -10	R superior border of hippocampus
					0.022 (3.71; 963)	-0.39	-37 -3 -27	L lateral border of amygdala/hippocampus
					0.052 (3.70; 780)	-0.39	-7 -28 -41	L anterior + posterior lobe of cerebellum

*determined from partial correlation coefficient analysis in SPSS

^#^with small volume correction for bilateral temporal lobes

**Figure 4:**
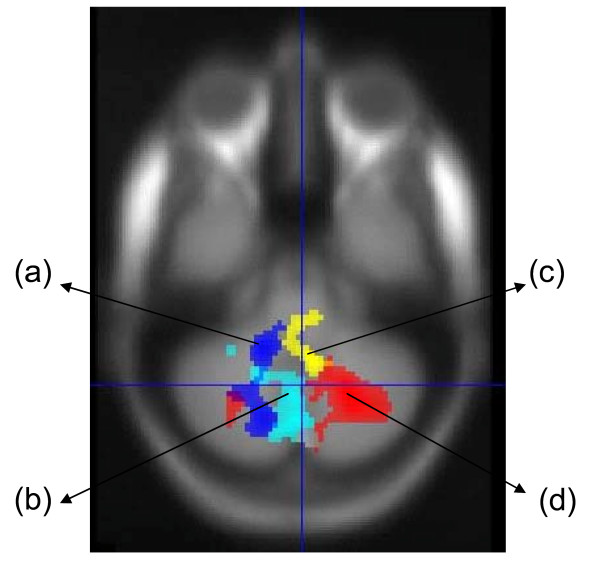
**Correlations with symptoms scores in cerebellar regions**. Negative correlations with suspiciousness/persecution score for (a) sentence completion, (b) verbal retrieval. Positive correlations with delusions score for (c) sentence completion, (d) verbal encoding. Maps thresholded at p = 0.005 uncorrected, extent threshold = 100 voxels. Left hemisphere shown on left of image

There was also a significant negative correlation between activation in the left posterior lobe of the cerebellum and the suspiciousness/persecution score for sentence completion versus rest (p = 0.002), and a more anterior and right sided region of the cerebellum was significantly positively correlated with the delusion score for the parametric contrast (p = 0.04), see Figure 4, Table 3.

#### Experiment 2: Encoding/retrieval task

The key finding from the encoding/retrieval task was a significant negative correlation between the suspiciousness/persecution score and activation in the bilateral MTL for the retrieval task (correct rejection versus experimental baseline activation, right p = 0.013, left p = 0.022), Figure 3b. There was also a negative correlation with activation in the left anterior and posterior lobes of the cerebellum (p = 0.052), Figure 3, Table 3, and a significant positive correlation between activation in the lentiform nucleus and the delusions score (p = 0.036).

For word classification versus experimental baseline there was a significant positive correlation between the delusion score and activation in the posterior lobe of the cerebellum (p = 0.001) (Figure 4), and a significant negative correlation between activation in the angular gyrus and the hallucinations score (p = 0.041), Table 3. There were no significant correlations with correct recognition versus experimental baseline.

## Discussion

Although previous studies have found associations between symptoms and functional imaging correlates in patients with established illness, the current study involved a large number of un-medicated high risk subjects experiencing psychotic symptoms and as such avoids potential contamination of findings by anti-psychotic medication and chronicity of disease. We performed these analyses by examining the effects of the presence of these symptoms on the neural responses to cognitive tasks. The tasks were not designed specifically to elicit symptoms, but were chosen to involve networks and cognitive processes known to be abnormal in patients with schizophrenia. The results indicated that symptom scores correlated with brain activation in a number of regions broadly consistent with the extant literature. Since in the analysis conducted the three symptom dimensions were modelled simultaneously within one design matrix, intersubject variability common to two or more symptom dimensions is not reflected in the correlation values. The values obtained therefore represent correlations specific to each symptom dimension which is not accounted for by either of the other two. However, due to the approach taken, we would like to state that since all high risk subjects were entered into the analysis, the correlation values may to some extent reflect the presence of symptoms as well as severity. As hypothesised there were significant correlations with the hallucination score and activation in the left lateral temporal cortex for the sentence completion task. For the encoding/retrieval task there were significant correlations with the suspiciousness/persecution score and activation in the bilateral MTL. Across both tasks there were significant correlations between delusions and suspiciousness/persecution scores and activation in the cerebellum, with different patterns of laterality.

The finding of an association between the hallucinations measure and activation in lateral temporal cortex is consistent with other reports linking this region to auditory hallucinations [9-11,14], and with cognitive neuropsychological theories of auditory verbal hallucinations regarding the misattribution of inner speech [1,34]. Although regions most typically associated with hallucinations are mid to posterior sections of the superior temporal gyrus, other studies have indeed reported associations with activation in the middle temporal gyrus [10,14,16]. The cognitive model most consistent with our task and results is that auditory hallucinations represent sub-vocal verbalisation which is not associated with the usual inhibition of auditory cortex that signifies self-production [1].

The memory task revealed strong negative correlations between the suspiciousness/persecution score and activation in bilateral MTL structures. This association was seen for correct rejection versus experimental baseline and not correct recognition. There is previous evidence however to indicate MTL regions are preferentially involved in correctly identifying new words (correct rejection) compared to correctly identifying old words (correct recognition) [35]. Previous literature has also implicated MTL regions in the formation of positive psychotic symptoms [3-5,36], and those that have examined delusional beliefs specifically also report decreased activation with increasing symptom severity [36]. There is also substantial evidence for structural abnormalities in this region in patients with established schizophrenia and high risk individuals [37-39]. The MTL, including the amygdala and hippocampus, are known to be involved in emotional, mnemonic and social processing [40-42] and dysregulation of these processes may contribute to the development of persecutory beliefs. For example, persecutory beliefs have been hypothesized to arise from a disruption in the processes mediating the formation and maintenance of normal social beliefs, and patients with persecutory beliefs have been shown to attend excessively to threat-related stimuli and to preferentially recall such information [43]. Impaired processing of threat-related stimuli has also been previously linked to dysfunction of the amygdala in paranoid patients with schizophrenia [44].

Although not part of our original hypothesis, we also found correlations between both suspiciousness/persecution and delusion scores and activation of the cerebellum across both of the tasks. Abnormal cerebellar function has been previously reported in the literature on the established illness [45] and in those at high genetic risk [26]. Further, it has been reported that patients with posterior lobe lesions have deficits which are not restricted to motor function, but also present affective disturbances, disinhibition, and psychotic symptoms [46,47]. The cerebellum has also been implicated in the 'cognitive dysmetria' model of schizophrenia where dysfunction in cortico-cerebellar-thalamic-cortical circuits are proposed to result in the abnormal synchrony or co-ordination of mental processing, ultimately resulting in the clinical features of the illness [48]. Although there are few imaging studies of patients with persecutory delusions, neuropsychological studies have repeatedly demonstrated that patients commonly attribute negative events to external sources [49]. Neurobiological theories have also directly implicated the cerebellum regarding deficits of 'internal monitoring' which are suggested, for example, to underlie the formation of delusions of alien control where the patient attributes their own actions to an external agent [19]. The current findings are therefore consistent with a growing literature implicating a role for the cerebellum in the formation of delusions.

The nature of the correlations in the cerebellum are however complex. The correlations are primarily right sided with regards to the delusions score, and primarily left sided with the suspiciousness/persecution score. It has been suggested that different types of delusions may differ pathophysiologically and there is an important role for emotion in the formation of paranoid/suspicious delusions via their effects on memory [49]. Following the model of brain function where emotion is predominantly associated with right sided cerebral regions, crossed cerebellar connections could mean that left sided cerebellar regions would be more involved in emotional/affective components of delusions, consistent with the present findings. Furthermore, since emotion is considered to be involved via its effect on memory processes, our MTL associations with the severity of suspiciousness/persecution also fits with this theory. We however recognise the speculative nature of these findings, particularly with regard to the paucity of other studies examining delusional beliefs. Further, in the group we have examined many had both hallucinations and delusions (including persecutory and non-persecutory) and it is therefore difficult to disambiguate individual components. That said, however, the fact that this was a study examining correlations with symptom scores reduces the chances of confounding effects by other symptoms. Studies directly manipulating experimental affective components in subjects with isolated symptoms may help clarify these results.

## Conclusion

In conclusion, these results support the view that hallucinations and delusions have differential pathophyisologies, that the lateral temporal cortex is associated with the experience of hallucinations, and that the MTL is associated with the production of, or perhaps the response to, suspiciousness/persecutory beliefs. The more novel finding of the involvement of cerebellar structures was seen across both tasks with both delusional and suspiciousness/persecution scores with differing patterns of laterality. That the current results are seen in un-medicated high risk subjects indicates these associations are not specific to the established illness and are not related to medication effects and hence may have relevance for the understanding of the biological basis of the disorder.

## Competing interests

The author(s) declare that they have no competing interests.

## Authors' contributions

HCW, VEG, JH, ES and DEJ contributed to analysis of the functional imaging data. AM contributed to statistical analysis. DGCO, ECJ and SML conducted clinical evaluations, conceived the original study, and participated in its design and co-ordination. All authors contributed to drafting of the manuscript and have read and approved the final version.

## Pre-publication history

The pre-publication history for this paper can be accessed here:


